# Supplementary material for: Color adjustment of brand logos for dark mode display
Source: PLoS One. 2026 Jan 21;21(1):e0339392. doi: 10.1371/journal.pone.0339392 (PMC12822999; doi:10.1371/journal.pone.0339392)
Supplement: Attachment — Submitted filename: Review Comments to the Author.docx [file pone.0339392.s004.docx]

Thank you for giving me the opportunity to review this manuscript. I think that the topic is interesting. The paper is well structured and easy to read. However, the paper has some flaws that authors must pay attention before the paper can get accepted:

**Introduction**

This section provides a thorough description of the "popularity of dark modes" and the "current research status of contrast, visibility and readability", but the transition from prior work to the research gap is not sufficiently clear. Please state the research gap and the innovative contribution more explicitly.

At present the manuscript mixes multiple motivations, including brand visual identity, interface comfort, and young user preferences, which makes it difficult for readers to distinguish the study’s theoretical contribution from its applied motivations. I recommend clarifying the hierarchy between the industry context and the theoretical background.

**Materials and Methods**

The Methods section describes the experimental workflow in reasonable detail; however, it lacks essential details on key control variables and measurement conditions.

The manuscript reports only the participant counts (e.g., 31 in the color adjustment experiment and 89 in the preference test) but lacks demographic and professional background details.

Although the ethics approval number is provided in the manuscript, I recommend moving this information to the beginning of the Methods section or the Participants subsection.

**Results**

The theoretical rationale and the validation procedure for the model need clearer articulation. In “Building a Color Adjustment Model for Dark Mode,” the authors use Kriging interpolation and CIELAB coordinates; please explain the theoretical motivation for these algorithmic choices and specify the error validation methods to make the modeling section more rigorous and assessable.

The data analysis methods appear somewhat weak. Please add details of the statistical tests and the significance levels to strengthen the credibility of the results.

**Discussion**

At present the Discussion section, particularly the beginning of “Findings and Insights,” moves directly into restating and comparing results, and it lacks a clear logical progression from experimental findings to theoretical interpretation to practical implications.

Although the manuscript states that “previous studies … focused on optimizing contrast,” it lacks comparative dimensions. For example, did prior work focus only on text readability? Has anyone attempted hue or saturation correction? Please expand the comparative discussion.

While the conclusion notes that the model could be extended to broader UI design, the outlook should be made more concrete. For example, discuss adaptation for animated icons, AR/VR environments, and differences between OLED and LCD displays; and potential contributions to sustainable branding systems or automated color correction systems. This would enhance the paper’s forward-looking value and citation potential.

**Limitations and Future Work**

The manuscript notes only that the experiments used 18 colors, a black background, and low-light conditions, but it lacks reflection on the diversity of samples and stimuli. For example, the participant pool appears to consist mainly of design students, which may limit generalizability, and cultural differences and device variability (display brands and gamut differences) are not considered.

At present the manuscript only makes a general suggestion to broaden the color range and lighting conditions. Please strengthen the Future Work section with actionable and forward-looking plans.

**Conclusion**

The conclusion is overly general, as in “user feedback was dominantly positive.” It should focus more tightly on the main findings supported by empirical evidence.

To strengthen the summary of theoretical and practical contributions, please add a sentence in the Conclusion that elevates the study’s value from a methodological contribution to a contribution to design theory.
