# Supplementary material for: Color adjustment of brand logos for dark mode display
Source: PLoS One. 2026 Jan 21;21(1):e0339392. doi: 10.1371/journal.pone.0339392 (PMC12822999; doi:10.1371/journal.pone.0339392)
Supplement: Attachment — Submitted filename: Reply to Reviewer Comments.docx [file pone.0339392.s006.docx]

**Reply to Reviewer Comments**

Title: Color Adjustment of Brand Logos for Dark Mode Display

We are deeply grateful to the reviewers for their insightful and constructive comments. We are encouraged that the overall framework, methodology, and contributions of our work were positively received. The reviewers’ thoughtful suggestions have been invaluable in helping us refine and improve the manuscript. In response, we have made careful revisions to better contextualize the study, enhance methodological transparency, and provide further detail where necessary. We believe these revisions have improved the clarity and academic rigor of the paper, and we sincerely hope that the updated manuscript meets the reviewers’ expectations.

**Responses to Editor:**

**Clarification Regarding the Use and Authorship of Fictitious Logos**

**Response:**

We would like to clarify that the logos presented in Figure 2 are entirely fictitious and do not correspond to any real-world brands or corporate identities. While the initial draft forms were generated using an automated tool, they were subsequently and fully reconstructed by members of our research team with formal training in design. This reconstruction involved adjustments to form, proportion, spacing, and color, ensuring that the final logos constitute original visual artifacts created solely for the purposes of this study. We have described this process explicitly in the manuscript to maintain full transparency regarding their authorship and originality.

If the editorial team has any remaining concerns or identifies any issues with these figures, we would be grateful for your guidance. We are fully prepared to revise or remove Figure 2, as well as any other figures containing fictitious logos, in accordance with your guidance.

**Responses to Comments from Reviewer 1**

**Overall Comment:**

Thank you for giving me the opportunity to review this manuscript. I think that the topic is interesting. The paper is well structured and easy to read. However, the paper has some flaws that authors must pay attention before the paper can get accepted.

**Response:**

We sincerely appreciate the reviewer for taking the time to evaluate our manuscript and for providing thoughtful and constructive comments. We are grateful that you found the topic interesting and the paper well structured and clear to read. We also acknowledge the concerns you raised and truly value your feedback. Your suggestions have been extremely helpful, and we are carefully addressing each point to improve the quality and clarity of the manuscript. Thank you again for your insightful review and for helping us strengthen our work.

**Comment 1-1:**

This section provides a thorough description of the "popularity of dark modes" and the "current research status of contrast, visibility and readability", but the transition from prior work to the research gap is not sufficiently clear. Please state the research gap and the innovative contribution more explicitly.

At present the manuscript mixes multiple motivations, including brand visual identity, interface comfort, and young user preferences, which makes it difficult for readers to distinguish the study’s theoretical contribution from its applied motivations. I recommend clarifying the hierarchy between the industry context and the theoretical background.

**Response 1-1:**

Thank you very much for your valuable feedback. We fully agree with your observation that the flow of the Introduction requires improvement. In response, we have revised this section to more clearly and convincingly articulate the research gap and the necessity of our study. Additionally, we reorganized the Introduction into subsections to ensure a clearer and more coherent structure for readers. We hope that the revised version reflects meaningful improvement and aligns better with your expectations.

✓ Please check the revised version of Introduction session.

**Comment 1-2:**

The Methods section describes the experimental workflow in reasonable detail; however, it lacks essential details on key control variables and measurement conditions.

The manuscript reports only the participant counts (e.g., 31 in the color adjustment experiment and 89 in the preference test) but lacks demographic and professional background details.

Although the ethics approval number is provided in the manuscript, I recommend moving this information to the beginning of the Methods section or the Participants subsection.

**Response 1-2:**

Thank you very much for your valuable feedback. Based on your suggestions, we have improved the Method section of the manuscript. Specifically, we added more detailed information about the participants, including their country of origin, and provided a clearer explanation of the recruitment process. In line with your recommendation, we also moved the IRB information to the participant subsection. We would be very grateful if you could review the revised section.

✓ Please check the revised version of Methods session.

**Comment 1-3:**

The theoretical rationale and the validation procedure for the model need clearer articulation. In “Building a Color Adjustment Model for Dark Mode,” the authors use Kriging interpolation and CIELAB coordinates; please explain the theoretical motivation for these algorithmic choices and specify the error validation methods to make the modeling section more rigorous and assessable.

The data analysis methods appear somewhat weak. Please add details of the statistical tests and the significance levels to strengthen the credibility of the results.

**Response 1-3:**

Thank you very much for your thoughtful comments. We agree that the previous version did not provide sufficient justification for the selection of certain metrics and methodologies. In response, we have added additional explanations regarding the use of the CIELAB color space and Kriging interpolation in this study. We hope that these revisions help to strengthen the overall logic and clarity of the manuscript. Thank you again for your valuable feedback.

✓ Please check the revised version of Results session.

**Comment 1-4:**

At present the Discussion section, particularly the beginning of “Findings and Insights,” moves directly into restating and comparing results, and it lacks a clear logical progression from experimental findings to theoretical interpretation to practical implications.

Although the manuscript states that “previous studies … focused on optimizing contrast,” it lacks comparative dimensions. For example, did prior work focus only on text readability? Has anyone attempted hue or saturation correction? Please expand the comparative discussion.

While the conclusion notes that the model could be extended to broader UI design, the outlook should be made more concrete. For example, discuss adaptation for animated icons, AR/VR environments, and differences between OLED and LCD displays; and potential contributions to sustainable branding systems or automated color correction systems. This would enhance the paper’s forward-looking value and citation potential.

**Response 1-4:**

We sincerely appreciate your thoughtful feedback and fully agree with your observation. You rightly pointed out that the previous Discussion section largely reiterated the Results and lacked sufficient depth. In response, we have substantially revised this section by conducting a more thorough comparison between our findings and existing literature, and by elaborating further on the practical implications of our study. We would be grateful if you could review the revised discussion.

✓ Please check the revised version of Discussion session.

**Comment 1-5:**

The manuscript notes only that the experiments used 18 colors, a black background, and low-light conditions, but it lacks reflection on the diversity of samples and stimuli. For example, the participant pool appears to consist mainly of design students, which may limit generalizability, and cultural differences and device variability (display brands and gamut differences) are not considered.

At present the manuscript only makes a general suggestion to broaden the color range and lighting conditions. Please strengthen the Future Work section with actionable and forward-looking plans.

**Response 1-5:**

Thank you very much for your thoughtful feedback. As you correctly noted, this study did not fully consider participants’ cultural or background diversity, and in the second experiment, display conditions were not strictly controlled. Although this was intentionally designed to examine the extensibility of the proposed model, we acknowledge that it may have limited the precision of the analysis. We recognize this as a limitation of our study and have explicitly addressed it in the revised manuscript. We would be grateful if you could review the updated section.

✓ Please check the revised version of Limitations and Future Work session.

**Comment 1-6:**

The conclusion is overly general, as in “user feedback was dominantly positive.” It should focus more tightly on the main findings supported by empirical evidence.

To strengthen the summary of theoretical and practical contributions, please add a sentence in the Conclusion that elevates the study’s value from a methodological contribution to a contribution to design theory.

**Response 1-6:**

Thank you for reviewing this part of the manuscript. We agree that the previous conclusion section was not sufficiently developed. Based on your feedback, we have revised it to present a more precise and substantial discussion of the study’s key insights and contributions. We would be grateful if you could kindly review the updated conclusion.

✓ Please check the revised version of Conclusion session.

**Responses to Comments from Reviewer 2**

**Overall Comment:**

This study gives a systematic, data-supported, and stable method. It helps designers and companies change brand logo colors well when they switch to dark mode. This method helps keep the brand look consistent, makes it comfortable for users to see, and makes sure it is visible.

**Response:**

Thank you very much for your positive and encouraging feedback. We truly appreciate your recognition of our attempt to propose a systematic and data-supported approach for adjusting brand logo colors in dark mode. We are grateful that you found the method valuable in maintaining brand consistency, enhancing visual comfort, and ensuring visibility. Your remarks reinforce the significance of this research, and we have further clarified these contributions in the revised manuscript to better communicate them to readers. Thank you again for your thoughtful evaluation.

**Comment 2-1:**

But there are still things to improve. The study is an early quantitative (number-based) study. As the authors said in the limitations section, it has weaknesses. These include a limited number of color samples, using only one simple background, and limited lighting conditions.

**Response 2-1:**

Thank you for your accurate and insightful comments. As this study is exploratory in nature and represents an attempt that has not been widely undertaken in the design field, we believe it holds meaningful value. Accordingly, our intention was to use a limited number of stimuli to clearly identify emerging tendencies rather than to generalize findings. To clarify the positioning of our work, we have added explicit statements in both the Limitations and Conclusion sections, emphasizing that this study is exploratory and in an early stage, while also acknowledging its limitations. We would greatly appreciate it if you could review the revised sections.

✓ Please check the revised version of Limitations and Future Work and Conclusion session.

**Comment 2-2:**

Also, a weakness the authors did not mention is that the test subjects were all very similar.

**Response 2-2:**

Thank you for your precise and thoughtful observation. As you rightly pointed out, this study was conducted only with young adult participants from South Korea. Considering that both color perception and logo recognition can be significantly influenced by cultural and age-related factors, the homogeneity of our sample indeed represents a limitation. We have acknowledged this in the manuscript and added a discussion suggesting that future research should include participants from diverse cultural and age groups to further clarify these differences. We would be grateful if you could review the revised section.

✓ Please check the revised version of Limitations and Future Work session.

**Comment 2-3:**

Later, the study used an online survey to get user feedback. This is not enough for visual feedback research. Usually, this kind of research uses physiological feedback surveys, like visual neuroscience and EEG. Future follow-up studies can first look for related research methods in other papers and use those as a basis for getting user feedback.

**Response 2-3:**

Thank you for this valuable observation. As you pointed out, subjective surveys have inherent limitations in producing definitive conclusions. We fully acknowledge this issue and have added a statement in the manuscript to clarify this limitation, along with a note that future studies may expand on this approach with more objective or complementary methods. We would be grateful if you could review the revised section.

✓ Please check the revised version of Limitations and Future Works session.

**Responses to Comments from Reviewer 3**

**Overall Comment:**

Authors present a study on developing a computational model to adjust the color in dark mode of brand logos originally designed for light mode user interfaces. First, Authors performed an experiment in a controlled environment to collect data on how a set of participants (Design-major students) manually adjusted artificially generated logos into their dark mode variants. Based on these data, Authors implemented a computational model for automatic color adjustment, which was then tested by a different set of participants on a less controlled experiment. According to their analyses, Authors state that the computational model was well received and that it could be applied across diverse usage contexts.

**Response:**

Thank you very much for your thoughtful summary and understanding of our work. Your concise description accurately reflects the intention and structure of our study. As you noted, our research consists of two stages: (1) a controlled experiment to observe manual adjustments of light-mode logos into dark-mode versions, and (2) the development and validation of a computational model using a separate participant group in a less controlled environment. We appreciate your recognition of our approach and conclusion that the model shows potential for application in broader contexts. In the revised manuscript, we have also clarified the study design, participant selection, and application scope to ensure that the contributions and limitations of our work are communicated more clearly.

**Comment 3-1:**

I found the topic of the study interesting and timely, given the recent rise in popularity of dark mode user interface and a personal preference for this mode. Overall, the article is well written in terms of grammar and spelling. Concerning the data collection during both experiments, I appreciated the fact that the study had a formal IRB approval, and a few important aspects were taken into consideration in trying to minimize bias; albeit I found not asking participants their preferred mode (light or dark) before the experiments an oversight. The construction of the computational model is also well detailed, and the provided code offers a reference implementation for interested Readers. Nevertheless, the scarce quantification of participant data, the lack of statistical rigor in the (few) conducted analyses, and the unavailability of the most crucial datasets for a perception-based model —i.e., participant-level data— severely hinder the manuscript assertions, and by consequence its publication. In addition, I found that there is a lack of discussion regarding current branding practices for the different modes. For instance, the Netflix logo achieves similar contrast ratios with the same colors in both light and dark modes (over 4.4:1), and its guidelines¹ require a minimum contrast ratio with any background (2.25:1). Finally, there are several presentational issues, including with Figures, which given the very visual-focused nature of the study, I found unfortunate.

**Response 3-1:**

Thank you very much for your valuable feedback. In response to your comment, we have added content to the manuscript describing how various companies, including Netflix and Spotify, address dark mode design. For the remaining points you raised in this section, we will provide more detailed responses in the relevant comments. We truly appreciate your insightful suggestions.

✓ Please check the revised version of Practical Design Implications session.

**Comment 3-2:**

PLOS journals require Authors to make available all data necessary to replicate study findings. This is not the case for the study at hand, as the related code and data uploaded to Zenodo only cover the computational model itself but not the data used to construct such model. There is no individual-level data on how each participant adjusted the logo colors in Experiment 1, which represent the very basis for the proposed perception-based adjustment model. There is no individual-level data on how each participant expressed their logo remembrance and preference in Experiment 2. There is no (anonymized) data regarding individual characteristics of participants. These data are crucial to be able to reproduce Authors’ results and to give support to their assertions and overall discussion in the manuscript. In addition, the code provided (in the form of a Jupyter notebook) should be better prepared as accompanying material to the manuscript in an international context. There are many comments in Korean, and there are also several warnings at stderr level at the end of the notebook.

**Response 3-2**:

Thank you sincerely for your valuable feedback. Based on your suggestion, we have revised the comments within the provided model code to be in Korean. In addition, to enhance data accessibility, we have shared the survey results from both experiments alongside the manuscript. We would be grateful if you could review these updated materials.

✓ Please check the revised version of Supporting Information.

**Comment 3-3:**

Many times, Authors’ statements are not backed by statistical analyses or summaries. For instance, in the section of “Trend of Color Adjustments for Dark Mode Display”, there is no quantification of the changes in L*, C*, and h*, only a textual description is given. In addition, it is not stated if these changes are significant or not. For instance, in line 247 Authors state «The process of adjusting logo colors on a black background resulted in bright colors becoming darker and dark colors becoming brighter, while the hue angle remained relatively stable.» However, this is not quantified, e.g., by giving the average difference in luminance and hue for the two groups (light and dark colors); no statistical test is conducted to verify if this luminance difference between the two groups is significant; and no table nor figure illustrate such differences. This is also important for changes in contrast ratios (mentioned in the Discussion, lines 424 and 429), which again, are not quantified nor tested for significance. Similar comments can be made for the other color dimensions.

**Response 3-3:**

Thank you for your important and constructive feedback. We fully agree with your point that providing only the original logo colors and the adjusted logo colors from the experiment was not sufficient. In response, we have added a visual plot to help readers intuitively observe and understand the changes in color. Furthermore, we conducted additional quantitative analyses of the LCh values to ensure that our findings go beyond descriptive statements and are presented with greater clarity and precision. We would be grateful if you could review the revised content.

✓ Please check the revised version of Building a Color Adjustment Model for Dark Mode session.

**Comment 3-4:**

In fact, there are only two statistical tests in the study, both of which regard Spearman rank correlation for Experiment 2 and both of which are —in my opinion—not adequate. The first test, on line 407, concerns proportions of logo remembrance and adjustment preference. The correlation is actually moderate (ρ = -.42) and the p-value is relatively close to α = .05. Nonetheless, testing the significance of overall proportions is not adequate for the question at hand (is logo remembrance associated with adjustment preference?). A better approach is to construct a 2×2 contingency table for participants, with “remembrance” (yes/no) and “preference” (non-adjusted/adjusted) and then conduct an association test (χ² or Fisher’s exact test). For the second test, on line 416, which concerns the correlation between color change (measured as the Euclidian distance) and logo preference (non-adjusted/adjusted), perhaps is better to conduct a point-biserial correlation or another test for dichotomous and continuous variables.

**Response 3-4:**

Thank you for providing feedback that we had not previously considered. In the original analysis, we treated the percentage responses for each stimulus as continuous data. However, after reviewing your suggestion, we agree that your proposed approach is more reliable. Accordingly, we revised our statistical method and reanalyzed the data. Using the chi-square test, we found that familiarity does influence preference for the model, though the effect size is relatively small. This revised analysis has been incorporated into the manuscript, and we would be grateful if you could review the updated section.

✓ Please check the revised version of Performance Test from Preference Survey session.

**Comment 3-5:**

Attention should be paid to PLOS One guidelines²on figures. In many cases the labeling font size is too small. In addition, most of the Figures should be (ideally) in vector format (e.g., EPS) or, if in raster format (e.g., TIFF), they should be a least 300 DPI.

**Response 3-5:**

Thank you for your thoughtful feedback regarding the quality of the figures. We carefully reviewed this point and have replaced all figures in the manuscript with high-resolution images of at least 300 DPI. We sincerely appreciate your attention to detail, and we would be grateful if you could review the updated figures in the revised version.

**Comment 3-6:**

In Tables 1 and 2, I suggest using white for the name of dark colors as the cell contrast is too low, especially for Dark Blue and Dark Purple. I also suggest a right justification of the numeric cells to ease comparisons, as well as separating the standard deviations in table 2 into their own columns for the same reason.

**Response 3-6:**

Thank you very much for your valuable and insightful feedback. In response to your suggestion, we have adjusted the text color in certain parts of the table to white to improve readability. We hope that the revised table enhances clarity and is now easier to interpret. We sincerely appreciate your careful attention to detail and your thoughtful guidance.

✓ Please check the revised version of Tables 1 and 2 session.

**Comment 3-7:**

In Fig. 4, I suggest annotating the dark mode images (A and B) with the respective HSV values of the Samsung logo, so Readers can have a quantitative notion of how color values change after an adjustment.

**Response 3-7:**

Thank you for your thoughtful feedback. As this figure serves as a representative illustration of the survey structure, we have revised it to clearly distinguish between the original and adjusted logos, rather than merely presenting their HSV values. We sincerely appreciate your constructive suggestion, which helped us improve the clarity and communicative value of the figure.

✓ Please check the revised version of 2. Related Works session.

**Comment 3-8:**

In Fig. 8, plot subtitles erroneously refer to the proportion of participants who preferred the adjusted logos as “ratio”. A ratio is a quantitative relation between two amounts, such as the contrast ratio between two colors.

**Response 3-8:**

Thank you for your valuable suggestion. In accordance with your feedback, we have replaced the original expression with percentage values. We would be grateful if you could review the updated figure.

✓ Please check the revised version of Figure 10.

**Comment 3-9:**

Supplemental Figures S1 and S2 are not original, based on a reverse image search. I invite Authors to double check if their supplemental figures respect PLOS One requirements concerning image licensing and copyright.

**Response 3-9:**

Thank you very much for your keen and insightful feedback. We appreciate you pointing out this overlooked aspect. Upon careful consideration, we recognized that the figure you mentioned does not meaningfully contribute to the core findings of the study, and therefore, we decided to remove it from the manuscript. We are grateful for your thoughtful observation, which helped us improve the clarity and focus of the paper.
